# Poster Session I - A76 TO EUS OR NOT? EVALUATING THE NECESSITY OF ENDOSCOPIC ULTRASOUND IN UNEXPLAINED BILIARY DUCT DILATION WITH NORMAL LIVER FUNCTION TESTS: A COMPREHENSIVE SYSTEMATIC REVIEW

**DOI:** 10.1093/jcag/gwaf042.076

**Published:** 2026-02-13

**Authors:** Z Alfaraj, R Mohanna, D M Rodrigues, L Hookey

**Affiliations:** Queen’s University, Kingston, ON, Canada; Queen’s University, Kingston, ON, Canada; Queen’s University, Kingston, ON, Canada; Queen’s University, Kingston, ON, Canada

## Abstract

**Background:**

Unexplained biliary duct dilation (CBD/PD) is commonly detected incidentally on imaging studies (US, CT, MRI), yet its clinical significance remains unclear. Elevated liver function tests (LFTs) typically suggest biliary pathology, while normal LFTs complicate diagnosis in non-jaundiced patients. Although endoscopic ultrasound (EUS) may provide valuable insights, its yield and cost-effectiveness in this group are not well established.

**Aims:**

This systematic review aims to evaluate the yield of significant EUS findings in non-jaundiced patients with unexplained biliary duct dilation and normal LFTs.

**Methods:**

We conducted a comprehensive literature search across multiple databases, including PubMed, Embase, Medline, Google Scholar, Scopus, the Cochrane Library, and Web of Science, from inception to October 2025. Two independent reviewers screened titles, abstracts, and full texts to identify studies reporting EUS findings in non-jaundiced patients with unexplained biliary duct dilation and normal LFTs. Studies with both normal and abnormal LFTs were included, with data specific to the normal LFT subgroup extracted when available.

**Results:**

Our review yielded 2,540 unique records, and 15 retrospective observational cohort studies were included in our analysis. These studies involved 1,442 non-jaundiced patients with normal LFTs who underwent EUS for unexplained biliary duct dilation. Malignancy was found in only 1.1% (16/1,442) of patients, primarily pancreatic cancer, which was more frequently observed with isolated pancreatic duct dilation. The pooled diagnostic yield for any pathology was 14.5% (209/1,442), with choledocholithiasis as the most common finding 7.4% (108/1,442). Other benign abnormalities included chronic pancreatitis (1.2%), sludge/microlithiasis (1.3%), diverticulum (0.9%), papillary stenosis (0.7%), and ampullary adenoma (0.6%). Factors associated with dilated biliary ducts included age over 65, female gender, Caucasian ethnicity, post-cholecystectomy status, and narcotic use. However, limited demographic data were available for patients with positive EUS findings.

**Conclusions:**

Our review indicates that EUS in non-jaundiced patients with unexplained biliary duct dilation and normal LFTs has a modest yield of positive findings at 14.5%, predominantly benign. The yield of critical findings, such as malignancy, is low at only 1.1%. Given these results, a cost-effectiveness analysis is crucial to guide clinical decision-making and optimize patient selection for EUS. Factors related to biliary duct dilation, such as patient age, history of cholecystectomy, and narcotic use, are important considerations. Additional research is needed to validate these findings and develop cost-effective strategies for EUS implementation.

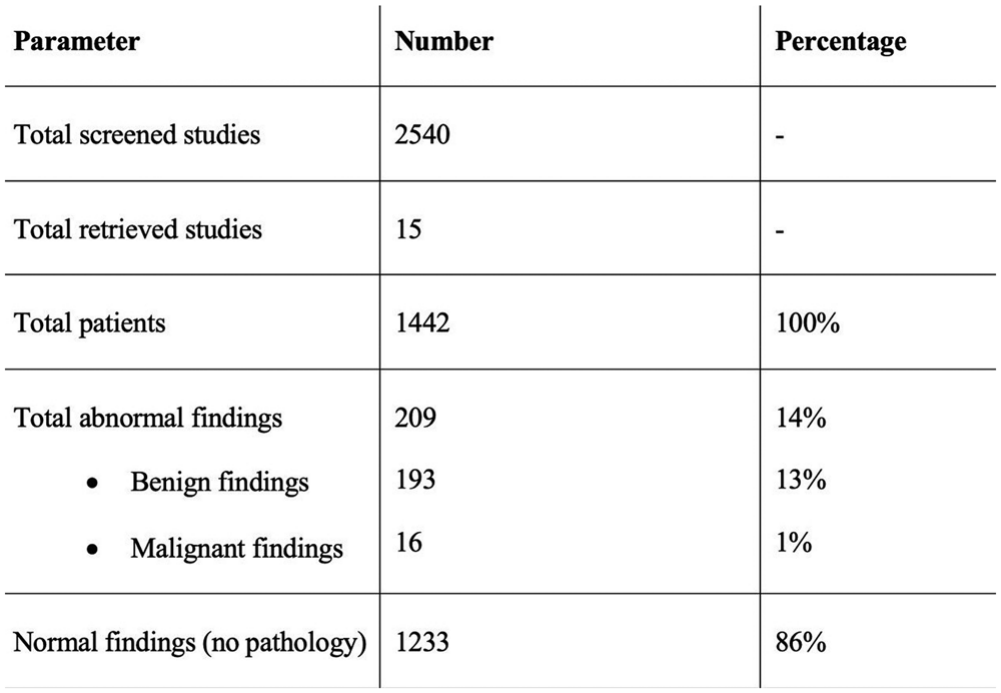

**Funding Agencies:**

None

